# Nicotinic Acid Receptor Abnormalities in Human Skin Cancer: Implications for a Role in Epidermal Differentiation

**DOI:** 10.1371/journal.pone.0020487

**Published:** 2011-05-31

**Authors:** Yira Bermudez, Claudia A. Benavente, Ralph G. Meyer, W. Russell Coyle, Myron K. Jacobson, Elaine L. Jacobson

**Affiliations:** Arizona Cancer Center and Department of Pharmacology and Toxicology, College of Pharmacy, University of Arizona, Tucson, Arizona, United States of America; Université de Technologie de Compiègne, France

## Abstract

**Background:**

Chronic UV skin exposure leads to epidermal differentiation defects in humans that can be largely restored by pharmacological doses of nicotinic acid. Nicotinic acid has been identified as a ligand for the human G-protein-coupled receptors GPR109A and GPR109B that signal through G_i_-mediated inhibition of adenylyl cyclase. We have examined the expression, cellular distribution, and functionality of GPR109A/B in human skin and skin derived epidermal cells.

**Results:**

Nicotinic acid increases epidermal differentiation in photodamaged human skin as judged by the terminal differentiation markers caspase 14 and filaggrin. Both GPR109A and GPR109B genes are transcribed in human skin and in epidermal keratinocytes, but expression in dermal fibroblasts is below limits of detection. Receptor transcripts are greatly over-expressed in squamous cell cancers. Receptor protein in normal skin is prominent from the basal through granular layers of the epidermis, with cellular localization more dispersive in the basal layer but predominantly localized at the plasma membrane in more differentiated epidermal layers. In normal human primary and immortalized keratinocytes, nicotinic acid receptors show plasma membrane localization and functional G_i_-mediated signaling. In contrast, in a squamous cell carcinoma derived cell line, receptor protein shows a more diffuse cellular localization and the receptors are nearly non-functional.

**Conclusions:**

The results of these studies justify future genetic and pharmacological intervention studies to define possible specific role(s) of nicotinic acid receptors in human skin homeostasis.

## Introduction

The skin functions as a metabolically active biological barrier protecting against external environmental insults. The maintenance of the skin barrier is critical as many dermatological diseases are characterized by defective barrier integrity resulting in a diminished protective capacity of the skin. Skin barrier formation is characterized by a process of homeostasis where dividing cells in the basal layer continually migrate toward the skin surface and undergo terminal differentiation and ultimately planned cell death to form the stratum corneum composed of terminally differentiated keratinocytes and other substances that form the barrier [1]. However, epidermal proliferation and differentiation must be carefully balanced since inadequate proliferation results in a thinning of the epidermal layer of skin and loss of barrier protection while increased proliferation without coordinate increased differentiation leads to disorders such as psoriasis and skin cancers [1].

A major environmental threat resulting in multiple alterations in skin structure and function is the exposure to ultraviolet light from the sun [2]. Chronic solar light exposure can ultimately lead to epidermal hyperproliferation with impaired differentiation resulting in actinic keratosis (AK) lesions and squamous cell carcinoma (SCC) of the skin. The main histological characteristic of SCC is substitution of normal matured epidermal keratinocytes with poorly differentiated atypical squamous cells [3]. Mudgil et al. have demonstrated in skin epidermal equivalents that transformed, poorly differentiated keratinocytes can be driven to differentiate in the presence of sufficient numbers of normal keratinocytes [4]. These observations suggest that treatments that could promote epidermal differentiation in photodamaged skin have the potential to restore the balance of proliferation and differentiation required for maintaining skin homeostasis and thus could counteract the development of AK lesions and SCC.

One of the pharmacological effects of nicotinic acid on photodamaged skin is to promote epidermal differentiation [5], raising the possibility that a nicotinic acid receptor may be involved. The receptor termed GPR109A (HM74A in humans and PUMA-G in mice), recently given the HGNC designation of NIACR1, was discovered in highly differentiated adipocytes, spleen, and immune cells [6], [7], [8]. GPR109A couples to G proteins of the G_i_ family, an inhibitory G protein, that upon ligand binding signals the decrease of adenosine 3′,5′-cyclic monophosphate (cAMP) production via the inhibition of adenylyl cyclase in a pertussis toxin-sensitive manner [6], [7], [8]. GPR109A is a member of a subfamily of G-protein-coupled receptors (GPCR) composed of GPR109A and GPR81 both of which are found in humans and rodents. GPR109B (also termed HM74) is another member of this receptor family that binds albeit with low affinity to nicotinic acid and is only present in humans. Nicotinic acid activates both GPR109A and GPR109B with EC_50_ values of 0.1 µM and 100 µM, respectively [9]. While it is considered unlikely that nicotinic acid is the endogenous ligand of these receptors, pharmacological doses of nicotinic acid acting through GPR109A mediate both desired anti-lipolytic effects as well as unwanted cutaneous vasodilatation effects associated with oral nicotinic acid therapy [6], [7], [8], [10], [11]. Since nicotinic acid-induced prostanoid formation responsible for skin flushing has been suggested to take place in dermal dendritic Langerhans cells, the expression of GPR109A in skin has been studied in this context [10], [12], [13], [14]; however, little is known about the functions of nicotinic acid receptors in other aspects of skin biology. Here we examine the expression, cellular distribution, and functionality of GPR109A and GPR109B in skin and skin derived cells. Our results show that squamous cell cancers have abnormal GPR109A/B expression, cellular distribution, and function, indicating that these receptors are involved in skin homeostasis.

## Materials and Methods

### Cell Culture

Normal human epidermal keratinocytes (NHEK, Lonza, Switzerland) were routinely cultured in EpiLife (GIBCO/Invitrogen) containing human keratinocyte growth supplement for less than 10 population doublings. Normal human diploid fibroblasts, CF3, (population doublings less than 30, Oklahoma Medical Research Foundation, Oklahoma City, OK), the established cell line of immortalized human epidermal keratinocytes, HaCaT, and Ras-transformed HaCaT, gifts from Dr. Norbert Fusenig (German Cancer Research Center, Heidelberg, Germany) [15], the human epidermoid carcinoma cell line, A-431 (American Type Culture Collection (ATCC), Manassas, VA), and the squamous cell carcinoma cell line, SCC-25 (ATCC) were routinely cultured in Dulbecco's Modified Eagle Medium (DMEM, Sigma Aldrich, St. Louis, MO) containing 10% fetal bovine serum (FBS, Hyclone/Fisher Scientific). All cells were kept in a humidified atmosphere containing 5% CO_2_ at 37°C.

### Reverse Transcriptase Polymerase Chain Reaction (RT-PCR)

Total RNA from adipose tissue, placenta, lung, and skin were purchased from Stratagene (Cedar Creek, TX) and 1 µg of each RNA was used to perform cDNA syntheses using the TaqMan Reverse Transcription kit from Applied Biosystems (Foster City, CA). PCR was performed using the following primers for GPR109A and GPR109B: GPR109A – forward – 5′ CACCACACAGACACACACCTCCTTGCTGG 3′, GPR109B – forward – 5′ CACCATACAGACACACGCCACTTTGCTGG 3′, and the reverse primer for both genes – 5′ CAGTGACATTACTCGATGCAACAGCCCAAC 3′ synthesized by Integrated DNA Technologies (IDT, Coralville, IA). Thermal cycling conditions were as follows: activation of DNA polymerase at 94°C for 1 min, followed by 25 cycles of amplification at 94°C for 1 min and 62°C and 68°C for 1 min each.

### Quantitative RT-PCR (qRT-PCR)

Total RNA from cultured cells was prepared using the RNeasy Mini Kit purification system (Qiagen, Valencia, CA) according to the manufacturer's instructions. Frozen achieved human normal skin biopsies, AKs, and SCCs were obtained from the project entitled “Chemoprevention of Skin Cancer Program Project Archived Samples for Additional Analyses, reviewed by the University of Arizona IRB (Tucson, AZ), Project Number 08-0201-04. The study from which the samples were obtained was conducted according to the Declaration of Helsinki Principles. Written informed consent was obtained from all subjects. Total RNA from skin tissue samples was prepared using the RNeasy Fibrous Tissue Mini Kit (Qiagen) following the manufacturer's protocol. cDNA synthesis was performed using the TaqMan Reverse Transcription kit (Applied Biosystems, Foster City, CA) according to manufacturer's instructions using random hexamers and 1 µg of total RNA. For TaqMan-based qRT-PCR expression profiling, 25 ng of each cDNA including total RNA from squamous cell carcinoma samples (Asterand, Detroit, MI) was added to the iTaq Supermix (BioRad, Hercules, CA) along with the TaqMan MGB probes according to the manufacturer's protocol (Applied Biosystems, Foster City, CA). qRT-PCR was performed as previously described [16]. Probes designed to specifically detect human GPR109A (TaqMan Gene Expression Assay Hs02341584_s1) and GPR109B (TaqMan Gene Expression Assay Hs02341102_s1) transcripts were purchased from Applied Biosystems. Real-time fluorescence monitoring was performed with the ABI Prism 7000 (Applied Biosystems). In human skin tissues, glyceraldehyde 3-phosphate dehydrogenase (GAPDH) and β-actin gene expression changed with the degree of malignancy compared to 18s rRNA, which remained constant. In cultured cells, GAPDH and β-actin expression remained constant when compared to 18s rRNA; therefore relative expression levels of the various transcripts were determined by comparison against the housekeeping genes, 18s rRNA for tissues and GAPDH for cultured cells. All expression measurements were performed in triplicate using at least three independently generated cDNA samples. Results are expressed as the mean ± standard error of the mean (SEM). Relative gene expression was calculated as described previously [17]. Alterations in gene expression were considered significant at 1.5-fold change and p≤0.05.

### Antibody Generation and Purification

For analysis of human GPR109A and GPR109B, a custom, commercial antibody was generated in rabbits using a synthetic peptide (RHHLQDHFLEIDKKNC) to generate antisera recognizing the N-terminus of GPR109A and GPR109B (Sigma-Genosys, Woodlands, TX). Animal maintenance and antibody production were performed by Sigma-Genosys according to the protocol reviewed and approved by the Sigma-Genosys Institutional Animal Care and Use Committee (IACUC), OPRR Assurance A4182-01; USDA Registration Number 93-R-283. Antisera were affinity-purified using Pierce's Aminolink Kit according to the manufacturer's protocol (Rockford, IL). The antibodies were validated by demonstrating that extracts from HeLa cells, which do not express the receptor, did not show positive immunoblots but extracts from HeLa cells (American Type Culture Collection, Manassas, VA) transfected with the nicotinic acid receptor did show positive bands at the expected migration position. Additionally, CF3 cells that did not show receptor expression by qRT-PCR also did not show positive immunoblots. Also, all immunoblot analyses included a control that showed that the peptide against which the antibodies were generated competed out the signal.

### Immunoblotting

SDS-PAGE and Western blot analyses were performed as described previously [18]. Adherent cell populations were lysed in ice-cold lysis buffer (150 mM sodium chloride, 50 mM sodium fluoride, 50 mM potassium phosphate, 40 mM sodium pyrophosphate, 500 µL Triton X-100, 100 µL SDS, 200 µL 1 M Tris-HCl pH 7.4 in water and 1 tablet of complete mini protease inhibitor cocktail, Roche Diagnostics, Indianapolis, IN) for 30 min at 4°C. Lysates were then centrifuged at 12,000 rpm for 30 min at 4°C. Protein concentration of the lysates was determined using the BCA™ Protein Assay Kit (Pierce, Rockford, IL) according to the manufacturer's instructions. Twenty micrograms of protein were added to 4× loading buffer (250 mM Tris pH 6.8, 8% SDS, 20% glycerol, 0.012% bromophenol blue, 4% β-mercaptoethanol), heated to 95°C for 5 min, electrophoresed in 12.5% SDS-polyacrylamide gels, and transferred to PVDF membranes (Amersham, Piscataway, NJ). All membranes were blocked for 1 h with 5% non-fat milk in Tris Buffered Saline (TBS) plus 0.1% Tween-20 (10 mM TBS-T, pH 7.4) and incubated overnight at 4°C in primary antibody or in primary antibody plus 1000 molar excess peptide (used to generate the antibody) relative to antibody, incubated in 5% non-fat milk TBS-T 24 h prior to usage. Membranes were incubated and developed in Immobilon™ Western Chemiluminescent HRP Substrate (Millipore, Billerica, MA) according to manufacturer's instructions. After initial blotting, membranes were reprobed for β-actin (Sigma-Aldrich, St. Louis, MO) to ensure even loading. Blots were scanned and analyzed with ImageJ version 1.39u software (NIH, Bethesda, MD). Values reported for target proteins were normalized to the blots' respective β-actin levels in at least three independent experiments.

### Immunocytochemistry (ICC)

CF3, NHEK, HaCaT, Ras-transformed HaCaT, A-431, and SCC-25 cells were grown on coverslips. For fixation, cells were rinsed twice with TBS, incubated in 4% formaldehyde in TBS for 30 min, and rinsed twice with TBS. To label the GPR109A/B receptors, endogenous biotin blocking was performed using NeutrAvidin™ Biotin-Binding Protein (Pierce) for 30 min at room temperature. Immediately after, the cells were incubated in 3% bovine serum albumin dissolved in TBS (BSA-TBS) blocking buffer for 1 h and incubated overnight at 4°C with primary antibody diluted in blocking buffer (1∶10). For peptide competition experiments, a 1000 fold excess of peptide was included. The following day, cells were washed 3 times for 5 min each with TBS. The blocking step was repeated and cells were incubated in 1∶25 Biotin-SP-conjugated AffiniPure Goat Anti-Rabbit IgG (Jackson ImmunoResearch Laboratories, West Grove, PA) for 30 min at 37°C. Slides were washed with TBS and incubated in 1∶25 Streptavidin Quantum Dot 605 Conjugates (Millipore) for 45 min at 37°C. After thorough washes with TBS, coverslips were mounted using mounting medium containing 90% glycerol and 10% TBS. The immunostained slides were placed at 4°C and visualized the following day under a fluorescence microscope.

### Immunohistochemistry (IHC)

Immunohistochemical detection of GPR109A/B was performed on paraffin-embedded sections from normal human skin biopsies. Archived material from a study conducted in accordance with the Declaration of Helsinki Principles reviewed by Integ Review Ethical Review Board, Austin, TX. All subjects read and signed an IRB-approved Consent Form. All participants, those administering the interventions, and those assessing the outcomes were blinded to group assignment. Paraffin-embedded sections were deparaffinized in xylene and hydrated in a graded series of alcohol, sections were immersed in citric acid in a water bath at 87°C for 20 min to enhance antigen retrieval. Sections were then rinsed in TBS and blocked in 3% BSA-TBS for 20 min. Immediately after blocking, sections were incubated in primary antibody in 3% BSA-TBS (1∶10) overnight at 4°C. For peptide competition experiments, a 1000 fold excess of peptide was included. The following day, sections were washed in TBS, blocked once again, and incubated in 1∶25 Biotin-SP-conjugated AffiniPure Goat Anti-Rabbit IgG (Jackson ImmunoResearch Laboratories, West Grove, PA) for 30 min at 37°C. Sections were rinsed with TBS and incubated in 1∶25 Streptavidin Quantum Dot 605 Conjugates (Millipore) for 45 min at 37°C. After thorough washes with TBS, coverslips were mounted using mounting medium containing 90% glycerol and 10% TBS. For peptide competition experiments, sections were washed in TBS and incubated in 1∶1000 FITC Goat Anti-Rabbit IgG (Jackson ImmunoResearch Laboratories) for 1 h at RT.

IHC analysis of caspase 14 and filaggrin involved analogous procedures using antibody SC5628 (Santa Cruz Biotechnology, Santa Cruz, CA) for caspase 14 and 3137-500 (Abcam, Cambridge, MA) for filaggrin. Quantification of antibody staining was performed as described previously [5].

### cAMP Assay

NHEK, HaCaT, Ras-transformed HaCaT, A-431, SCC-25, and CF3 cells were seeded at a density of 25,000 cells in 35 mm culture dishes. Two days later, culture medium was removed and replaced with culture medium containing ±100 ng/mL pertussis toxin (incubated overnight), ±10 µM forskolin in the presence or absence of nicotinic acid at different concentrations for 1 h prior to testing. Except for dose response studies, the final concentration of nicotinic acid used was 100 µM. Measurement of intracellular cAMP levels was performed using cyclic AMP Complete EIA Kit (Assay Designs, Ann Arbor, MI) per manufacturer's instructions. Experiments were repeated three times with independent cell cultures. Data are presented as mean ± SEM from three separate experiments.

## Results

### Nicotinic acid promotes epidermal differentiation in photodamaged human skin

**Figure 1:**
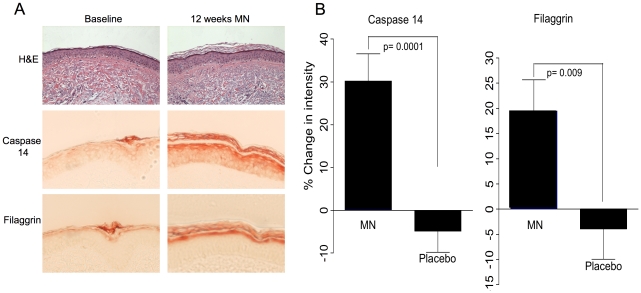
Nicotinic acid promotes epidermal differentiation in photodamaged human skin. Tissue arrays of skin biopsy samples from a clinical study of the effects of myristyl nicotinate (MN) in human subjects with photodamaged skin [5] were stained for the terminal differentiation markers caspase 14 and filaggrin. **Panel A:** An example of a biopsy sample at baseline and 12 weeks of MN treatment stained with H&E, and immunostaining for caspase 14 or filaggrin. **Panel B:** Quantification of staining for the placebo (n = 27) and MN treated (n = 31) groups for caspase 14 and filaggrin. Students t-test was used to compare placebo and MN treated groups and p values are shown.

Chronic UV exposure leads to impaired epidermal differentiation and increased epidermal proliferation that can result in actinic keratosis lesions and squamous cell cancers of the skin [19]. We have previously concluded that myristyl nicotinate (MN), a prodrug that delivers nicotinic acid to skin, promotes epidermal differentiation with associated enhanced skin barrier function in chronically photodamaged skin as judged by changes in epidermal and stratum corneum thickness, decreased rates of transepidermal water loss and increases in minimal erythemal dose [5]. To examine effects of MN on differentiation more directly, two epidermal differentiation markers, caspase 14 and filaggrin [20] were assessed in subjects with skin photodamage in a placebo controlled clinical trial [5]. Fig. 1A shows an example of biopsy samples stained at baseline and after 12 weeks of topical application of a formulation containing 5% MN and Fig. 1B shows quantification of caspase 14 and filaggrin in subjects treated with placebo or MN formulations. The presence of MN results in an increase relative to placebo of approximately 30% for caspase 14 and 20% for filaggrin. The placebo formulation substituted myristyl myristate for MN, ruling out any effects of myristyl alcohol that is generated as nicotinic acid is liberated from the prodrug.

### Nicotinic acid receptor genes are expressed in normal human skin

**Figure 2:**
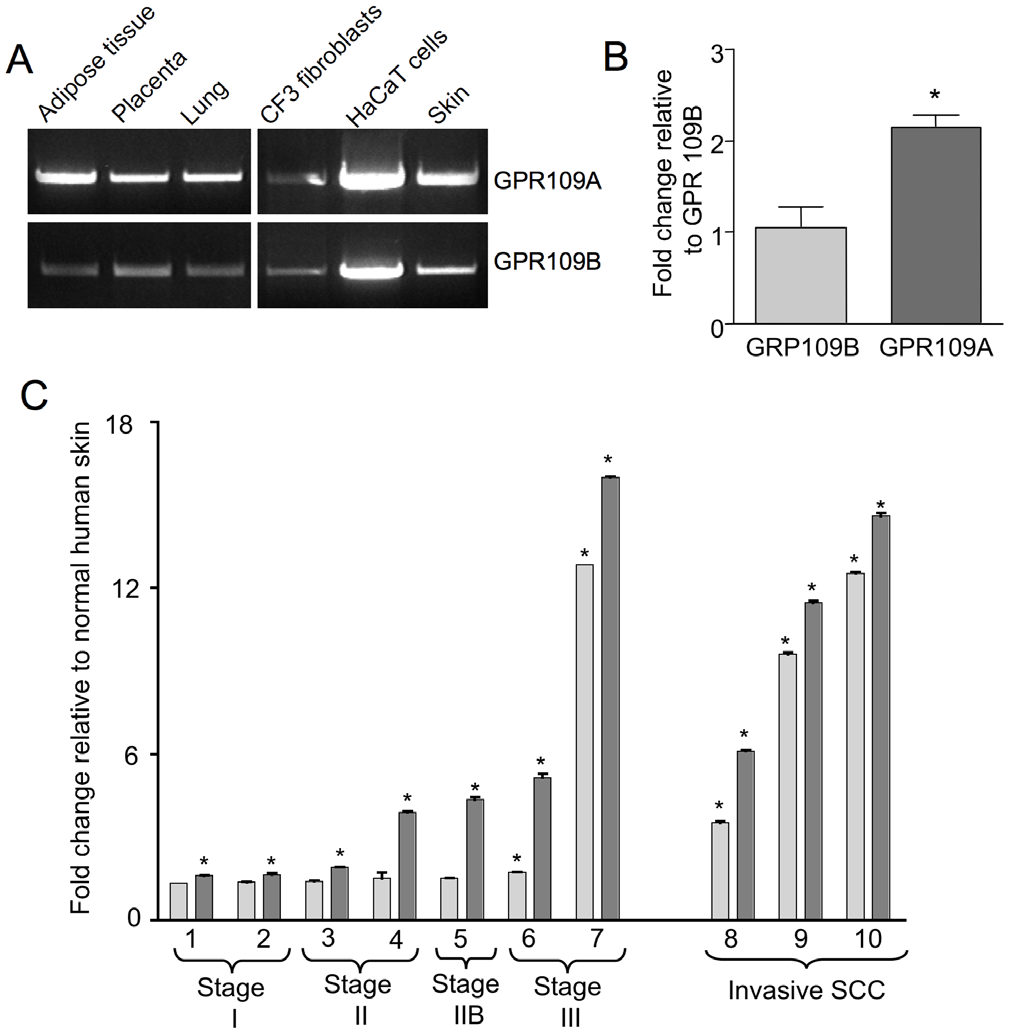
GPR109A and GPR109B are expressed in human tissues and cells including skin. **Panel A:** RT-PCR was performed on total RNA from adipose tissue, placenta, lung, and skin tissues and cells using primers specific for GPR109A (top row) and GPR109B (bottom row) as described in Materials and Methods. **Panel B:** qRT-PCR was performed on total RNA from six different normal human skin tissues using probes specific for GPR109A (dark grey column) and GPR109B (light grey column) receptors as described in Material and Methods. Students t-test was used to compare GPR109A to GPR109B, * p≤0.05. **Panel C:** qRT-PCR analyses were performed on total RNA from ten human squamous cell carcinoma tissues using probes specific for GPR109A (dark grey columns) and GPR109B (light grey columns) receptors. Results represent the mean ± SEM. Students t-test was used to compare to normal human skin, * p≤0.05.

The promotion of epidermal differentiation by nicotinic acid led us to characterize the nicotinic acid receptors in human skin as a putative nicotinic acid target. Genes encoding both high affinity (GPR109A) and low affinity (GPR109B) forms of the nicotinic acid receptor have been described previously and the expression of these genes has been reported in skin [6], [12], [21] but very minimal characterization has been reported. PCR analysis revealed expression of genes encoding nicotinic acid receptors in adipose tissue, placenta, and lung (Fig. 2A), tissues previously shown to express both receptor forms [6], [7], [8], [10], [12]. Fig. 2A also shows that transcripts for both high and low affinity forms of the receptor were readily detected in human skin and HaCaT cells. Very little signal was detected in CF3 dermal skin fibroblasts (the bright partial signal shown for GPR109A likely represents contamination from the HaCaT lane as it was not observed in repeat experiments). The relative levels of expression in human skin examined by qRT-PCR show that the gene encoding the high affinity form of the receptor is expressed at approximately 2.2 fold higher level than the low affinity form (Fig. 2B).

### Nicotinic acid receptor genes are over-expressed in squamous cell skin cancers

The expression of both GPR109A and GPR109B relative to that of normal skin was examined in squamous cell cancers (SCC) of different stages of progression (Fig. 2C). Stage I SCC samples show a slight increase in the mRNA expression of GPR109A, whereas GPR109B mRNA expression is not significantly elevated. In stage II and IIB tumors, GPR109A mRNA expression is elevated approximately 3-fold while GPR109B expression again remains similar to that of normal skin. In stage III and in unstaged tumors graded as “invasive SCC”, both receptor expression profiles are elevated up to 16-fold relative to normal human skin tissue. In general, increased expression of the GPR109 genes increases with the stage of cancer progression.

### Nicotinic acid receptor genes are transcribed and translated in skin cell lines

**Figure 3:**
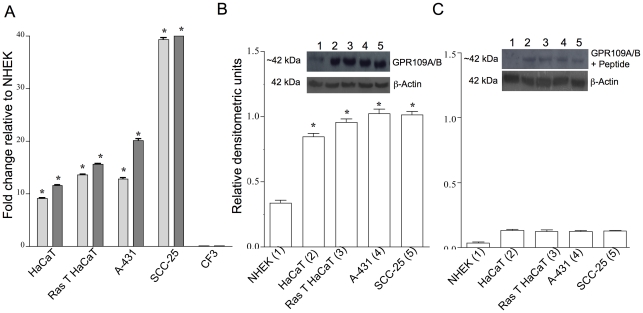
GPR109A/B genes are transcribed and translated in human skin cells. **Panel A:** qRT-PCR was performed on total RNA from normal human epidermal keratinocytes (NHEK), immortalized human epidermal keratinocytes (HaCaT), immortalized Ras-transformed human epidermal keratinocytes (Ras-transformed HaCaT), human epidermoid carcinoma cells (A-431), squamous cell carcinoma cells (SCC-25), and human diploid fibroblasts (CF3), using probes specific for GPR109A (dark grey columns) and GPR109B (light grey columns) receptors. Students t-test was used to compare to NHEK, * p≤0.05. **Panels B and C:** Protein Expression of GPR109A and GPR109B in Human Skin Cells. Cell extracts from NHEK (lane 1), HaCaT (lane 2), Ras-transformed HaCaT (lane 3), A-431 (lane 4), and SCC-25 (lane 5) were subjected to SDS-PAGE and Western immunoblot analyses using an antibody against GPR109A/B pre-incubated in the absence (panel B) or presence (panel C) of 1000-fold excess peptide against which the antibody was generated relative to purified antibody. β-Actin was used as a loading control for Western immunoblot analyses. One representative blot is shown of three independent experiments. The relative densities were quantified using ImageJ. Graphical representation shows the average densitometric units of three independent experiments. Students t-test was used to compare to NHEK, * p≤0.05.

Various skin cell lines were examined by qRT-PCR for GPR109A/B gene expression and the results were compared relative to normal primary human epidermal keratinocytes (NHEK). Immortalized epidermal keratinocytes (HaCaT cells), tumorigenic epidermal keratinocytes (Ras-transformed HaCaT), an epidermoid vulva carcinoma-derived cell line previously reported to contain nicotinic acid receptors (A-431), and a human squamous cell carcinoma cell line (SCC-25) express mRNA for both receptors (Fig. 3A). Over-expression relative to NHEK is observed up to 40-fold in skin cancer cells as compared to NHEK (Fig. 3A). In contrast, expression of nicotinic acid receptors in dermis-derived normal human diploid fibroblasts (CF3) is undetectable.

To determine if nicotinic acid receptor transcripts are translated, we examined cell extracts for GPR109A/B protein using an anti-peptide antibody developed and characterized in our laboratory (See Materials & Methods). This antibody targets a sequence near the N-terminus common to both receptors and does not differentiate between the highly homologous GPR109A and GPR109B proteins. Western blot analyses show that nicotinic acid receptor protein was detected in all cell lines examined at the expected size for the two nicotinic acid receptors (∼42 kDa) (Fig. 3B). The peptide used to generate the antibody effectively competed the antibody signal, demonstrating antibody selectivity (Fig. 3C). The amount of protein relative to β-actin is higher in the immortalized, transformed, and malignant cell lines examined compared to NHEK.

### Nicotinic acid receptors in human skin are present in the epidermis and hair follicle

**Figure 4:**
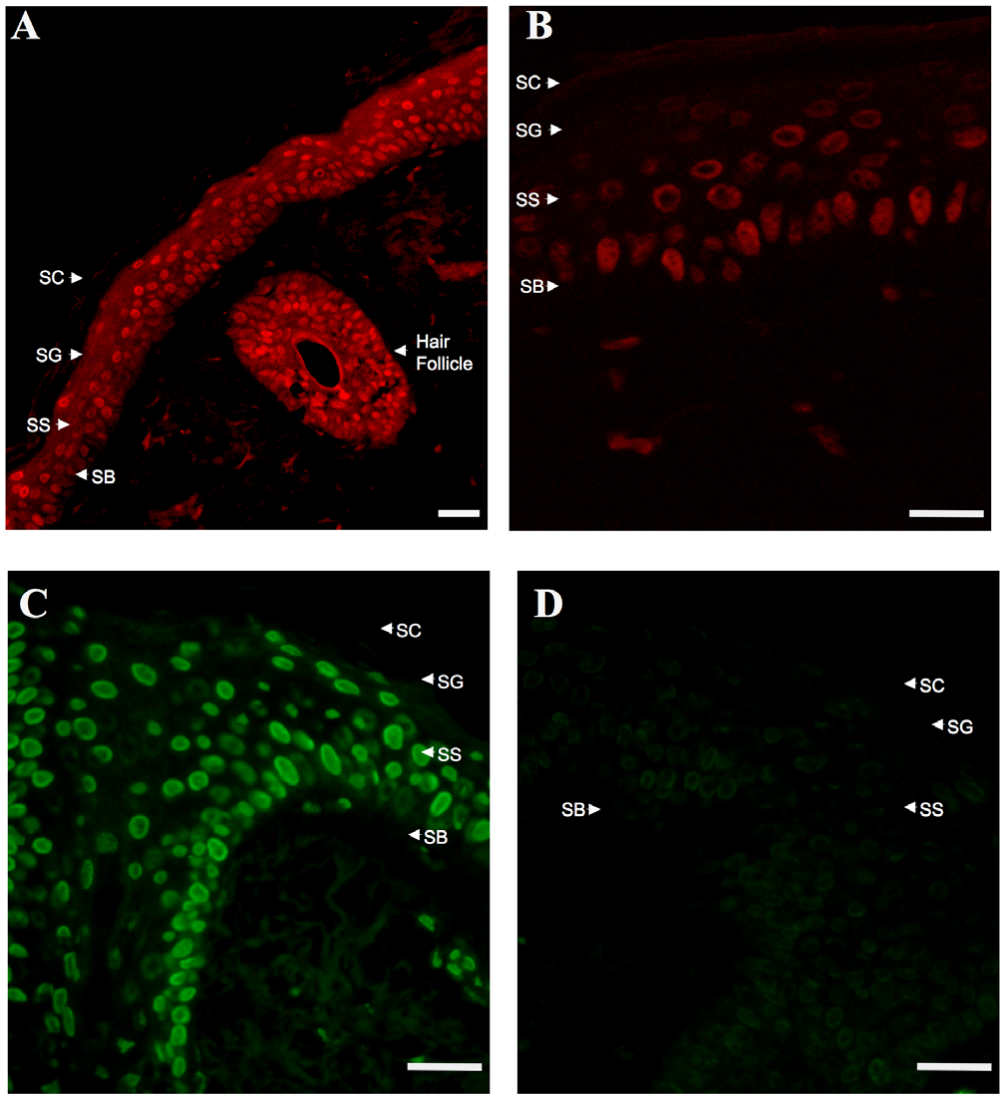
Localization of GPR109A/B protein expression in human skin. Immunohistochemistry (IHC) analyses were performed on paraffin-embedded human skin sections utilizing antibody against GPR109A/B. Panels A and B utilized Streptavidin Quantum Dot 605 Conjugates for detection. Panels C and D used FITC Goat Anti-Rabbit IgG for detection. **Panel A:** Representative immunostaining sample shown at 200× magnification. **Panel B:** Representative IHC sample shown at 400× magnification. **Panels C and D:** Representative IHC samples shown at 400× magnification in the absence (Panel C) or presence (Panel D) of competition with peptide used to generate the antibody. Abbreviations: SC, stratum corneum; SG, stratum granulosum; SS, stratum spinousum; SB, stratum basale. Size marker represents 2 microns.

To determine the cellular localization of GPR109A/B in human skin, immunostaining analyses were performed. We observed that GPR109A/B are expressed from the basal layer through the spinous and granular layers of the epidermis and in the hair follicles of normal human skin (Fig. 4A). Higher magnification (Fig. 4B) reveals that GPR109A/B expression appears to be more evenly distributed throughout the cell in the basal cell layers of the epidermis and shows a more peripheral distribution in the spinous and granular layers where keratinocytes are in later stages of terminal differentiation. An alternative fluorescent tag, Fluorescein isothiocyanate (FITC), for detecting anti-peptide antibody binding is used in Fig. 4C to demonstrate that peptide used to generate the antibody effectively blocked binding in these tissues (Fig. 4D).

### Nicotinic acid receptor cellular localization varies among cultured epidermal cells

**Figure 5:**
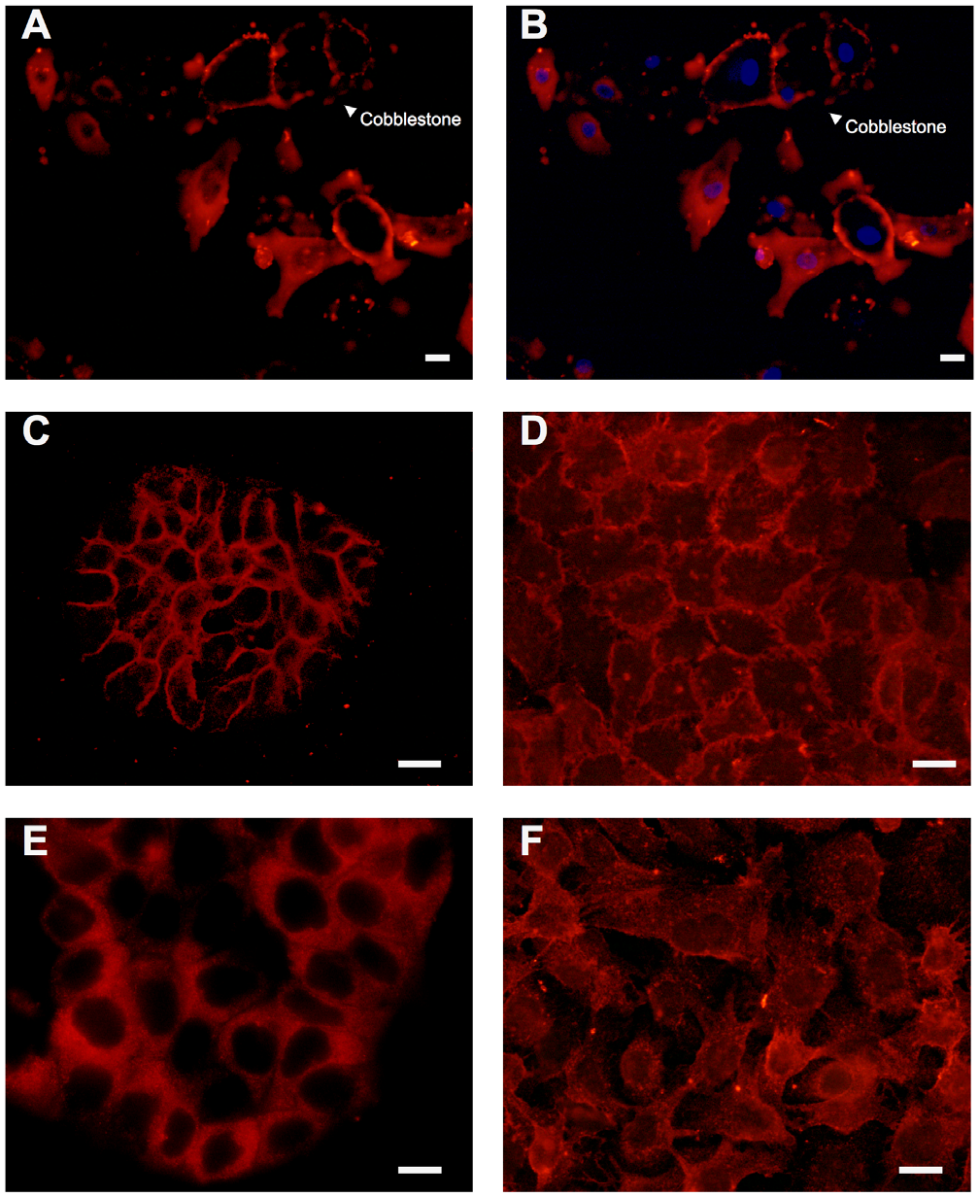
Localization of GPR109A and GPR109B protein expression in cultured normal and malignant human skin cells. **Panels A and B:** Immunocytochemistry (ICC) analyses utilizing an antibody against GPR109A/B were performed on NHEK shown in red and merged with nuclear DAPI staining in blue. Panel A shows staining using the GPR109A/B antibody and Panel B shows staining in the presence of 1000 fold excess of peptides used to raise the antibody. **Panel C:** HaCaT, **Panel D:** Ras-transformed HaCaT, **Panel E:** A-431, **Panel F:** SCC-25. For all panels, magnification was 400× and size marker represents 10 microns.

NHEK cells were stained using the anti-peptide antibody (Fig. 5A) and peptide competition blocked the staining (Fig. 5B). Staining appears on the periphery of aggregates of cells forming “cobblestone structures” while isolated cells appear to have a more diffuse pattern of labeling (Fig. 5A). In HaCaT cells, GPR109A/B expression is mainly localized to the periphery (Fig. 5C). In contrast, in Ras-transformed HaCaT (Fig. 5D), A-431 (Fig. 5E), and SCC-25 (Fig. 5F) the plasma membrane localization is accompanied by labeling throughout the cytoplasm. Receptor expression in skin fibroblasts, CF3, is undetectable (data not shown).

### Normal keratinocytes demonstrate a functional G protein-coupled nicotinic acid receptor signaling through G_i_ whose function is diminished in skin cancer cells

**Figure 6:**
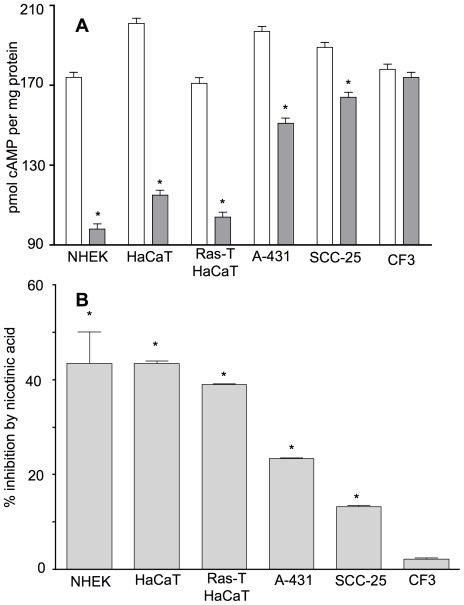
GPR109A and GPR109B are functional in normal human keratinocytes but are defective in malignant cells. **Panel A:** NHEK, HaCaT, Ras-transformed HaCaT, A-431, SCC-25, and CF3 cells were treated in the presence of 10 µM forskolin (open columns) or 10 µM forskolin and 100 µM nicotinic acid (grey columns) for 1 h followed by measurement of intracellular cAMP levels. Data are from three independent experiments and show forskolin-induced cAMP production relative to cellular protein. **Panel B:** The percent inhibition by nicotinic acid is shown for each cell line. For both panels A and B, Students t-test was used to compare cAMP produced without nicotinic acid to that produced with nicotinic acid, * p≤0.05.

Most characterizations of the functionality of nicotinic acid receptors have been studied in cells that do not inherently express the receptors but into which the receptor genes are transfected and over-expressed. In our studies, we examined intrinsic receptor functionality by measuring the ability of nicotinic acid to inhibit forskolin-stimulated cAMP formation in the absence of heterologous expression. Under these conditions, the relative contribution of nicotinic acid sensitive cAMP formation is less than that in transfected cells. Forskolin treatment elicited cAMP formation in all of the cells studied with an accumulation of 170–200 pmol cAMP per mg protein in a 1 h incubation and the presence of 100 µM nicotinic acid inhibited the forskolin-induced cAMP production differentially in the various cell types (Fig. 6A). The relative degree of inhibition in the various cell lines by nicotinic acid is shown in Fig. 6B. Inhibition of cAMP formation by nicotinic acid through the intrinsic receptors was approximately 44% in NHEK cells, 43% in HaCaT and 39% in Ras-transformed HaCaT cells. In contrast, cAMP stimulated by forskolin was significantly reduced in the tumor-derived cells. Nicotinic acid inhibited only 23% in A-431 cells and 13% in SCC-25 cells. Forskolin treatment elicits significant cAMP accumulation in CF3 cells but nicotinic acid fails to inhibit cAMP formation, consistent with the absence of nicotinic acid receptor expression observed in dermal fibroblasts (Fig. 3A).

**Figure 7:**
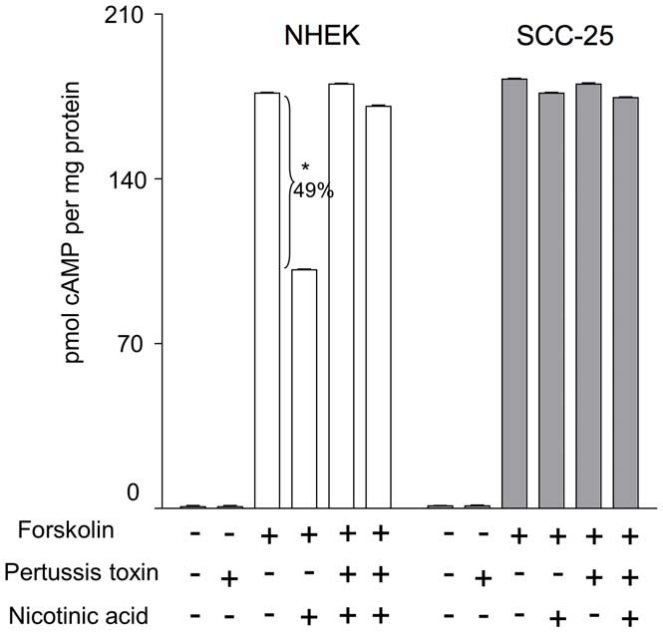
GPR109A/B couple through Gi in normal keratinocytes while SCC-25 cells are insensitive to pertussis toxin and nicotinic acid. NHEK (open columns) and SCC-25 (grey columns) cells were pretreated with or without 100 ng/mL pertussis toxin overnight and stimulated with 10 µM forskolin in the presence or absence of 100 µM nicotinic acid and intracellular cAMP levels were measured. Data represent the mean ± SEM from three independent experiments calculated as forskolin-induced cAMP relative to cellular protein. Students t-test was used to compare to cAMP produced without nicotinic acid to that produced with nicotinic acid, * p≤0.05.

To determine whether the response to nicotinic acid is signaling through the inhibitory G_α_ subunit and to further examine receptor functionality in SCC-25 cells, cAMP production in NHEK and SCC-25 was examined in the presence of pertussis toxin, a known inhibitor of the heterotrimeric G_i_ protein subunit (Fig. 7). Pertussis toxin completely abolished nicotinic acid-mediated inhibition of forskolin-stimulated cAMP formation in NHEK cells. In contrast, the response to nicotinic acid is very small in SCC-25 cells in the absence or presence of pertussis toxin. These data indicate that the nicotinic acid receptor(s) are signaling via G_i_ and further demonstrate that the receptor has greatly reduced functionality in SCC-25 cells.

**Figure 8:**
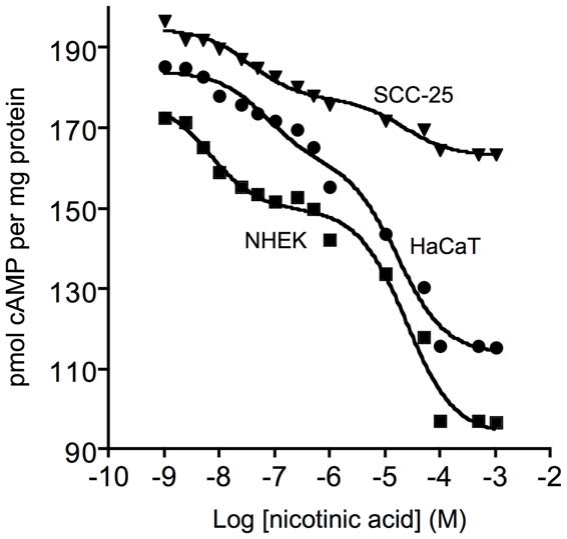
Effect of increasing concentrations of nicotinic acid on forskolin stimulated cAMP levels. NHEK (squares), HaCaT (circles), and SCC-25 cells (triangles) were treated with different concentrations of nicotinic acid the effects on cAMP levels were determined. Representative data are shown with curve fitting lines using a two site-binding model. The R^2^ values for the curve fitting were NHEK (0.985), HaCaT (0.988) and SCC-25 (0.987). EC_50_ values for GPR109A and GPR109B, respectively, calculated by curve fitting of data from multiple experiments were: NHEK (6.9 nM and 25 µM); HaCaT (72 nM and 17 µM); SCC-25 (36 nM and 22 µM).

To characterize receptor function further, we compared dose-dependent inhibition by nicotinic acid of forskolin-induced cAMP production in NHEK, HaCaT, and SCC-25 cells. Fig. 8 shows representative data and curves that fit the data to a two-site binding model predicted for binding to high affinity and low affinity receptors. Data from multiple experiments were fit to a two-site model to calculate EC_50_ values for receptor affinity and relative receptor abundance was assessed by the magnitude of reduction of cAMP generation by nicotinic acid. The data was a close fit to a two-site model for each cell line with R^2^ values for fit to the data points ranging from 0.985 to 0.988. NHEK cells showed evidence of a significant abundance of both GPR109A and GPR109B with EC_50_ values of 6.9 nM and 25 µM, respectively (R^2^ value of 0.985). Compared to NHEK, HaCaT cells showed a reduced abundance of GPR109A with lower affinity (EC_50_ of 72 nM) but a similar abundance and affinity of GPR109B (EC_50_ of 17 µM) (R^2^ value of 0.988). Compared to NHEK, SCC-25 cells showed a greatly reduced abundance of GPR109A and GPR109B, with calculated EC_50_ values of 36 nM and 22 µM (R^2^ value of 0.987). The EC_50_ value for the endogenous GPR109A in NHEK is lower than that reported previously but the other values for both GPR109A and GPR109B are similar to the previously reported EC_50_ values for GPR109A and GPR109B in cells transfected with the recombinant receptors or from *in vitro* studies reported to be approximately 100 nM and 100 µM, respectively [9]. These data further support the conclusion that nicotinic acid receptors have greatly reduced functionality in the tumor-derived SCC-25 cells.

## Discussion

Pharmacological doses of nicotinic acid have long been known to profoundly affect blood lipoproteins by decreasing low-density lipoproteins (LDL) and very-low density lipoproteins (triglycerides) and increasing high-density lipoproteins (HDL) [22], [23]. More recent studies have demonstrated pharmacological effects of nicotinic acid in skin. Nicotinic acid can prevent both UV-induced skin tumors and immune suppression in mice [24]. In humans, nicotinic acid promotes epidermal differentiation in photodamaged skin as assessed by increased epidermal and stratum corneum thickness, enhanced barrier function, and increased minimal erythemal dose [5]. We report here additional evidence that nicotinic acid enhances epidermal differentiation as measured by it effect on the terminal differentiation markers caspase 14 and filaggrin (Fig. 1).

Studies of the pharmacological effects of nicotinic acid have focused on nicotinic acid receptors GPR109A/B in humans [9]. These receptors have been mainly studied in the context of effects on blood lipoproteins and associated skin flushing side effects of oral nicotinic acid therapy [12]. While prior studies have surveyed human tissues or cell lines for GPR109A mRNA expression including skin [12], [21], [25], we describe here a more detailed characterization of the nicotinic acid receptors in human skin and skin-derived cells using qRT-PCR, Western blot, immunochemistry, and pharmacological analyses. Our studies show that GPR109A/B genes are transcribed and translated in skin keratinocytes and human skin epidermis (Figs. 2, 3, and 4A) but that expression in dermal fibroblasts is below limits of detection.

Studies reported here provide several pieces of evidence that support an in depth investigation of the hypothesis that GPR109A/B is involved in the differentiation-promoting effects of nicotinic acid in photodamaged human skin. First, the pattern of GPR109A/B expression in the epidermal layer of skin shows a more diffuse cellular localization in basal keratinocytes and a more peripheral membrane localization in areas of the epidermis undergoing active differentiation (Fig. 4B), supporting the possibility that the receptor is brought to the cell surface as keratinocytes undergo differentiation. Similar results are seen for normal keratinocytes in culture where isolated cells show a more diffuse localization but “cobblestone” clusters of cells beginning to differentiate show a more peripheral localization (Fig. 5A).

Second, endogenous GPR109A/B is functional in normal human keratinocytes in culture (Figs. 6, 7, 8). Most other studies that have examined nicotinic acid receptors have used transfected cells not inherently expressing GPR109A/B, although a previous study has reported that the epidermoid cell line A-431 highly expresses GPR109A and GPR109B receptors [25]. Even though this cell line is not of skin origin, we included it in the studies described here (Fig. 6). Our results corroborate the over-expression data of the previous study. Our studies indicate that the endogenous GPR109A has a higher affinity for nicotinic acid in NHEK cells compared to results of other investigators using transfected cells and isolated membranes but the affinity of GPR109A in HaCaT and GPR109B in both NHEK and HaCaT is similar to that reported both for transfected cells and isolated membranes [22]. For SCC-25 cells, the affinity of GPR109A is reduced compared to NHEK and the abundance of both receptors is greatly reduced.

Third, our results indicate that abnormalities in GPR109A/B transcription and function develop as progressive skin damage leads to differentiation defects seen in squamous cell skin cancers. The degree of over-expression of the GPR109A gene increases as a function of disease progression in skin (Fig. 2C) and cultured SCC-25 cells also significantly over-express the genes encoding the receptors compared with normal keratinocytes (Fig. 3A). Further, SCC-25 cells show an abnormal cellular localization (Fig. 5F) and contain a nearly non-functional receptor (Figs. 6, 7, 8). The severe reduction of functional cell surface receptors in SCC-25 cells may be related to the observed over expression of the genes, where increased mRNA levels represent an attempt to compensate for the lack of functional protein on the cell surface. The epidermoid carcinoma cell line, A-431, also shows reduced receptor function.

Our report of the loss of functional nicotinic acid receptors in SCC-25 cells is of interest with regard to a previous study of the potential role of GPR109A in colon cancer. Thangaraju *et al*. have characterized the expression of GPR109A in colon tissues and found expression in the lumen-facing apical membrane of colonic and intestinal epithelial cells. However the receptor is silenced in human colon cancers, in a mouse model of colon cancer, and in colon cancer cell lines [26]. They also show that GPR109A mediates the tumor suppressive effects of butyrate, a putative ligand of GPR109A in the colon, suggesting that the GPR109A acts as a tumor suppressor in the colon [26]. These data, taken together with our data, suggest that a functional GPR109A/B may be important in maintaining a differentiated state in epithelial cells and that cancer cells may utilize different mechanisms to generate a non-functional nicotinic acid receptor to avoid differentiation signals mediated by the receptor. While butyrate is proposed as a putative endogenous ligand of the receptor in the colon, the ketone body ß-hydroxybutryate has been proposed as an endogenous ligand in tissues [22]. ß-hydroxybutyrate would represent a logical ligand in skin as it accumulates under conditions such as fasting or starvation, where it could function to support skin barrier function under stress conditions.

In summary, the increased epidermal differentiation in nicotinic acid-treated human skin may involve nicotinic acid receptor-mediated signaling pathways, although other mechanisms may contribute to the effects of nicotinic acid on photodamaged skin. Both forms of the receptor are expressed in human skin, appearing mainly in the epidermis but are not present in the dermis. The altered expression of the genes encoding GPR109A/B, the abnormal pattern of cellular distribution, and impaired functionality of the receptor in squamous cell carcinoma cells suggest that progression of skin damage leading to receptor defects could provide a mechanism for squamous cell cancers to avoid differentiation signals. However, the observation that poorly differentiated keratinocytes can be driven to differentiate in the presence of sufficient numbers of normal keratinocytes [4] raises the possibility that treatments that could promote epidermal differentiation in photodamaged skin could restore the balance of proliferation and differentiation required for maintaining skin homeostasis and thus may counteract the development of AK lesions and SCC. In that context, nicotinic acid receptors could be potential targets for skin cancer prevention. The studies reported here provide sufficient evidence to justify genetic and pharmacologic interventions to define whether and how the nicotinic acid receptor family may function in promoting differentiation of photodamaged skin.
